# Probing infectious disease by single-cell RNA sequencing: Progresses and perspectives

**DOI:** 10.1016/j.csbj.2020.10.016

**Published:** 2020-10-21

**Authors:** Geyang Luo, Qian Gao, Shuye Zhang, Bo Yan

**Affiliations:** a Shanghai Public Health Clinical Center, Fudan University, Shanghai, China; b Shanghai Public Health Clinical Center and Key Laboratory of Medical Molecular Virology (MOE/NHC/CAMS), Shanghai Medical College and School of Basic Medical Sciences, Fudan University, Shanghai, China

**Keywords:** ACE2, Angiotensin-Converting Enzyme 2, ARDS, acute respiratory distress syndrome, ATAC-seq, Assay for Transposase-Accessible Chromatin using sequencing, BCR, B cell receptor, CEL-seq, Cell Expression by Linear amplification and Sequencing, *CLU*, clusterin, COVID-19, corona virus disease 2019, CRISPR, Clustered Regularly Interspaced Short Palindromic Repeats, CytoSeq, gene expression cytometry, DENV, dengue virus, FACS, fluorescence-activated cell sorting, GNLY, granulysin, GO analysis, Gene Ontology analysis, HIV, Human Immunodeficiency Virus, IAV, Influenza A virus, IGLV/LJ/LC, Immune globulin light V/J/C/ region, IGHV/HD/HJ/HC, Immune globulin heavy V/D/J/C/ region, ILC, Innate Lymphoid Cell, LIGER, Linked Inference of Genomics Experimental Relationships, MAGIC, Markov Affinity-based Graph Imputation of Cells, MARS-seq, Massively parallel single-cell RNA sequencing, MATCHER, Manifold Alignment To CHaracterize Experimental Relationships, MCMV, mouse cytomegalovirus, mcSCRB-seq, molecular crowding single-cell RNA barcoding and sequencing, MERFISH, Multiplexed, Error Robust Fluorescent In Situ Hybridization, MLV, *Moloney Murine Leukemia Virus*, MOFA, Multi-Omics Factor Analysis, MOI, multiplicity of infection, PBMCs, peripheral blood mononuclear cells, pDCs, plasmacytoid dendritic cells, *PLAC8*, placenta-associated 8, SARS-CoV-2, severe acute respiratory syndrome coronavirus 2, SAVER, Single-cell Analysis Via Expression Recovery, sci-RNA-seq, single-cell combinatorial indexing RNA sequencing, scRNA-seq, single cell RNA sequencing technology, seqFISH, sequential Fluorescent In Situ Hybridization, smart-seq, switching mechanism at 5′ end of the RNA transcript sequencing, SPLit-seq, split pool ligation-based tranome sequencing, STARTRAC, Single T-cell Analysis by RNA sequencing and TCR TRACking, STRT-seq, Single-cell Tagged Reverse Transcription sequencing, t-SNE, t-Distributed stochastic neighbor embedding, TCR, T cell receptor, TSLP, thymic stromal lymphopoietin, UMAP, Uniform Manifold Approximation and Projection, UMI, Unique Molecular Identifier, 3C, Chromosome Conformation Capture, Single-cell RNA sequencing, Infectious diseases

## Abstract

The increasing application of single-cell RNA sequencing (scRNA-seq) technology in life science and biomedical research has significantly increased our understanding of the cellular heterogeneities in immunology, oncology and developmental biology. This review will summarize the development of various scRNA-seq technologies; primarily discussing the application of scRNA-seq on infectious diseases, and exploring the current development, challenges, and potential applications of scRNA-seq technology in the future.

## 1 Introduction

Single-cell studies have greatly expanded our knowledge of cell subsets diversity and individual cell heterogeneity in organisms. The primary applications of these studies include single-cell analysis of transcriptome [1], DNA methylation [2], chromatin accessibility [3], chromatin interactions [4], histone modifications [5], histone marks [6], spatial transcriptome [7] and more. This review mainly focuses on scRNA-seq characterizing single-cell transcriptomes.

scRNA-seq technologies have greatly improved during the last decade and are widely used in immunology, tumor biology, developmental biology and other fields. Commercial scRNA-seq platforms, such as the *10X Genomics* and *BD Rhapsody*, have automated single cell lysis, RNA extraction, cDNA reverse transcription and library construction [8], [9], [10]. These advancements in simplifying the methodology and improving the cost-efficiency have allowed scRNA-seq to become one of the most popular and powerful life science and biomedical research tools available [11], [12].

## 2 The development of scRNA-seq technology

**Fig. 1:**
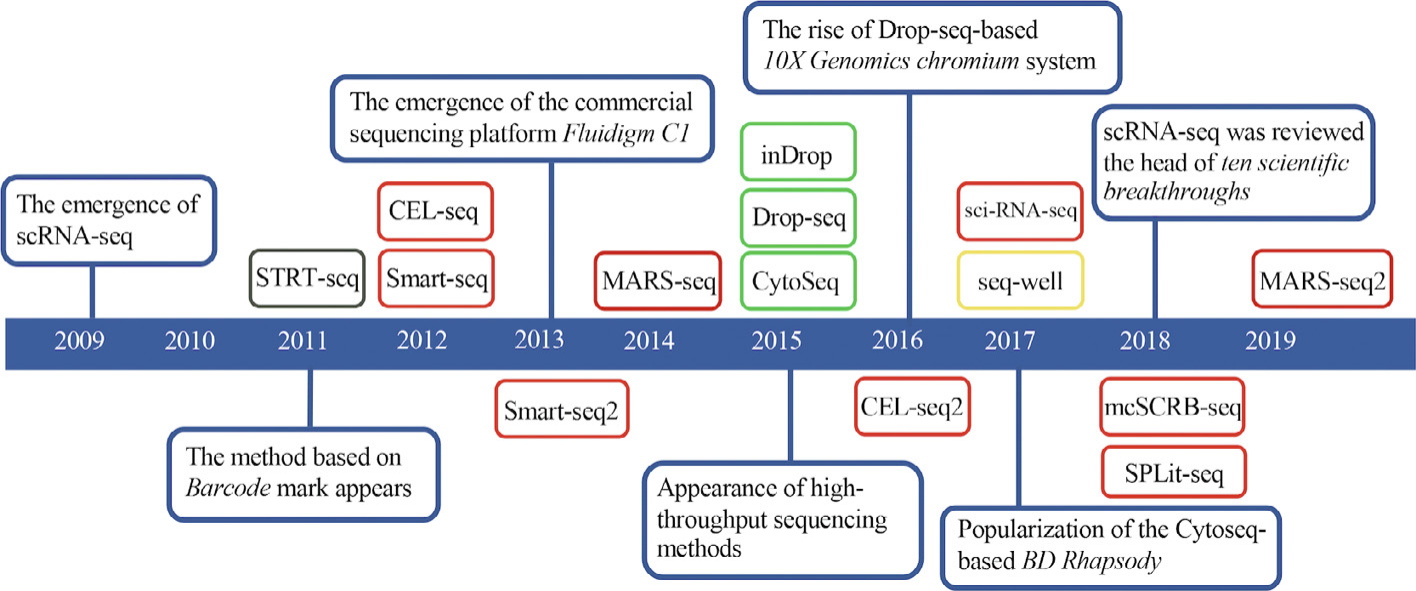
The blue boxes represent significant events in the history of scRNA-seq development. The remaining color boxes are abbreviations of various technologies. The black box represents microfluidics-based technology. The red boxes represent plate-based technology. Green boxes represent microdroplet-based technology. Yellow box represents nanowell-arrays-based technology. (For interpretation of the references to color in this figure legend, the reader is referred to the web version of this article.)

Tang et al. developed the first scRNA-seq method in 2009 [13], [14]. In 2011, Islam et al*.* furthered the technology by applying barcode labeling and developing single-cell tagged reverse transcription sequencing (STRT-Seq) which made high-throughput scRNA-seq possible [15]. Subsequently, with the continuous improvement in cell separation and nucleic acid amplification processes, novel scRNA-seq technologies surged (Fig. 1). These new advances included plate-based linear amplification and sequencing (CEL-seq) [16], combinatorial indexing-based single-cell combinatorial indexing RNA sequencing (sci-RNA-seq) [17], microdroplets-based inDrop [18], and Drop-seq [19].

Before the rise of high-throughput technologies, plate-based methods were the mainstream scRNA-seq platforms, which includes CEL-seq, CEL-seq2, Massively parallel single-cell RNA sequencing (MARS-seq) and MARS-seq2 [12], [20]. The emergence of unique molecular identifier (UMI) improved the quantification of mRNA contents by using random code for labelling individual mRNA strands [21]. Using UMI allows the investigator to distinguish the original template from the amplified sequence derived from the cDNA or library amplification [11], [21]. UMI counting has been incorporated in many scRNA-seq methods, including CEL-seq, CEL-seq2, MARS-seq, MARS-seq2, Drop-seq, inDrop, etc. [11], [22], [23], [24], [25].

CEL-seq is based on linear amplification and uses a unique barcode primer to obtain reverse-transcription in a single tube, which decreases sequencing errors [16]. CEL-seq2, the improved version of CEL-seq, is based on microfluidic chips on *Fluidigm’s C1* platform, giving it higher sensitivity, lower costs and less labor [26]. MARS-seq was previously the main large-scale method for scRNA-seq in immune profiling [27]. It uses fluorescence-activated cell sorting (FACS) of single cells into multi-well plates and subsequent automated processing, which leads to a significant increase in throughput and reproducibility [28]. MARS-seq2 was developed for index FACS sorting (recording the levels of surface marker for each sorted single cell) and based on the MARS-seq approach. The combination of FACS and scRNA-seq technology ensures MARS-seq2 can record both single-cell surface markers and transcriptomes, which is particularly useful for characterizing rare cell populations [20].

Other revolutionary platforms have emerged since 2015, such as Drop-seq and inDrop [18], [19]. Drop-seq uses UMI and barcodes to mark mRNA from individual cells, which then facilitates pooled sequencing from multiple cells. However, this technique can only detect a limited number of genes (5000 at best) per cell. Currently, Drop-seq is more popular as it is a high-throughput platform for discovering new cell types, construction of cell differentiation trajectory, molecular mapping of differentiation process, embryonic development and more. [29], [30], [31], [32].

Another commonly used scRNA-seq methods is Smart-seq2 [11], [22], which enables detecting higher number of genes, about 9000 per cell [11]. Although the number of cells assayed in each experiment is fewer, it is useful when dealing with samples containing limited cell numbers for traditional RNA sequencing, such as circulating tumor cells, early embryonic cells and some laboratory unculturable microorganisms [33]. Smart-seq2 also avoids the 3′ bias for most sequencing methods [11]. It uses MLV (*Moloney Murine Leukemia Virus*) reverse transcriptase which prefers to choose full-length cDNAs as substrates for its terminal transferase activity [34]. Special primer design also ensure identical primers for cDNA synthesis, which helps keep the PCR amplification efficiency consistent [34]. The combination of attributes listed above ensures the synthesis of full-length cDNA with the Smart-seq2. All exons of each transcript can be detected by Smart-seq2, which endues Smart-seq2 with the capability to detect alternative splicing [34], [35]. The Smart-seq3 has recently emerged as an upgraded version of Smart-seq2 with 5′ UMI for even more effective sequencing [36].

Due to a significant reduction in sequencing costs, the number of cells detected by scRNA-seq has increased from 10^2^ to 10^6^ per assay, and continues to grow. Downstream bioinformatic analytical tools have also been rapidly developing such as dimensionality reduction and its visualization techniques. These include t-SNE (t-Distributed stochastic neighbor embedding) and UMAP (uniform manifold approximation and projection) [37]. Personalized analysis tools included pseudotime, gene ontology (GO) analysis and STRING database network analysis and others. With the improvement of scRNA-seq technology and cost-efficiency at such a rapid pace, the ability to assess the cellular heterogeneity in life science and biomedical research by scRNA-seq will only increase with time. In the next section, the application of scRNA-seq in infectious disease studies will be discussed, using examples of severe acute respiratory syndrome coronavirus 2 (SARS-CoV2), human immunodeficiency virus (HIV), influenza A virus (IAV), cytomegalovirus, dengue virus (DENV), flavivirus, *Mycobacterium tuberculosis*, *Trypanosoma brucei*, *Salmonella, Toxoplasma gondii* and helminth.

## 3 Applications of scRNA-seq in infectious disease

### 3.1 Immune atlas study

#### 3.1.1 Identifying novel immune cell subtypes

**Fig. 2:**
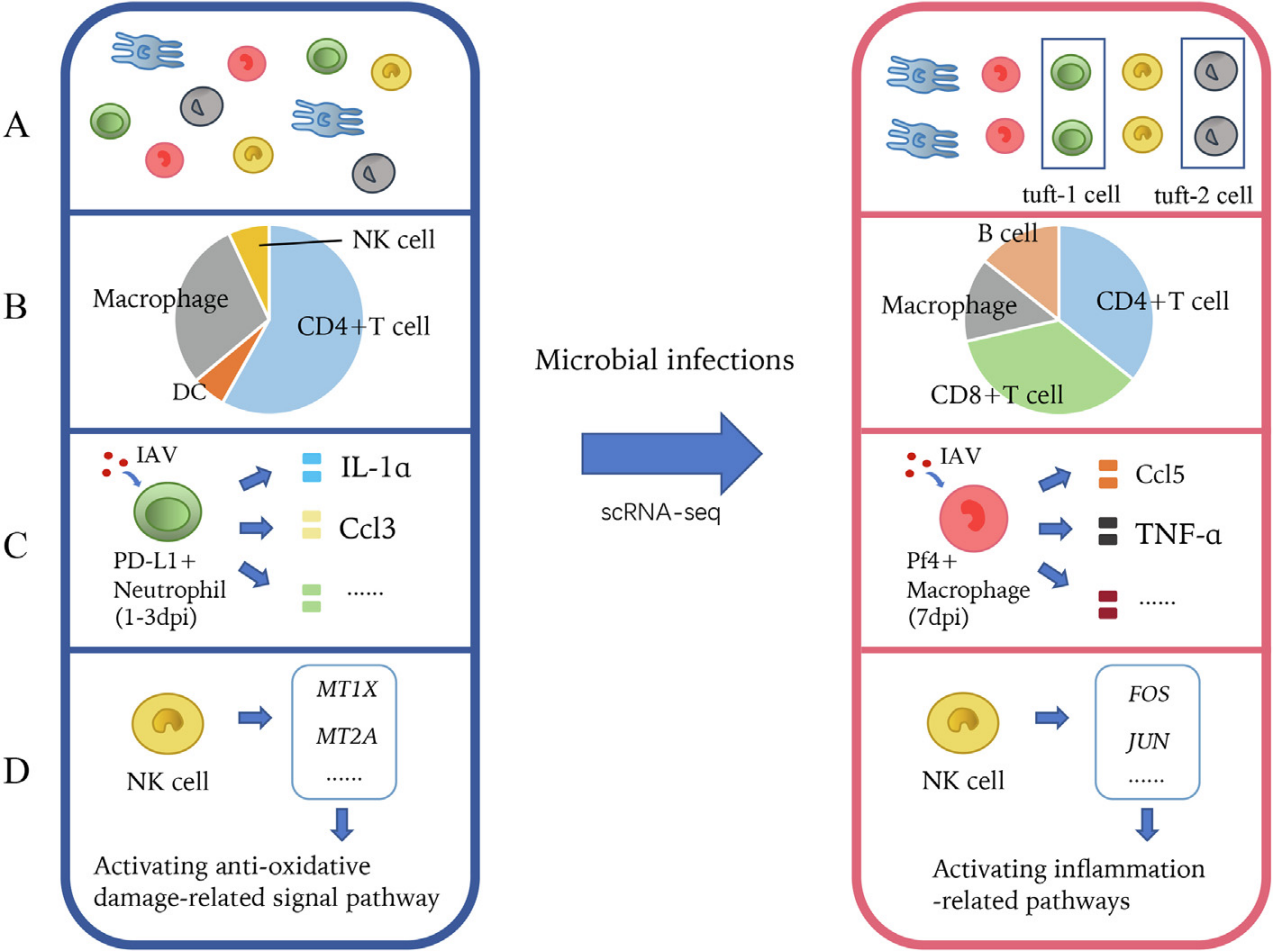
Immune atlas study. (A) Identifying novel immune cell subtypes; (B) Detecting immune cell landscape during infection; (C) Detecting changes of inflammatory responses; (D) Identifying immune signaling pathways for differentially expressed genes during infection.

When facing various infectious pathogens, heterogeneous immune cells are involved in various important biological processes, such as pathogen recognition, killing and antigen-presentation. For example, macrophages can be divided into various tissue-resident subtypes whose transcriptomes are significantly different. The identification of novel immune cell subgroups and understanding of their molecular characteristics, kinetics and functions during the infection process will greatly facilitate our understanding of both infectious disease mechanisms and the development of subsequent treatment strategies. To illustrate our point, based on bulk transcriptome analysis, it was determined that helminth infection induced *Trpm5*^+^ tuft cells expressed both neuronal and inflammation-related genes [38]. However, tuft cells could be subdivided into two subsets, tuft-1 and tuft-2, by scRNA-seq. Tuft-1 cells were mainly related to neuronal development, while tuft-2 cells primarily express thymic stromal lymphopoietin (*TSLP*) and other inflammation-related genes [39] (Fig. 2A). Using scRNA-seq and T cell receptor (TCR) clonal analysis, Waickman et al. report that clonal expanded T cells have unique transcriptional characteristics. A group of memory-precursor CD8^+^ T cells expressing CD38 molecules was defined in this study [40]. Similarly, it was demonstrated that CD4^+^ T cells latently infected with HIV could differentiate into two very different cell subtypes. The transcriptional level of HIV virus and the number of genes transcribed in type 1 cells were lower than those in type 2 cells, and type 1 cells were more difficult to be activated [41]. Macrophage subtypes have been identified in TB patients via scRNA-seq [42]. By utilizing the high-throughput and low-cost scRNA-seq technique called Seq-Well, Gierahn et al. identified three pulmonary macrophage subsets from TB patients. All these macrophages preserved the feature of TLR7/8 pathway activation, which is involved in the intracellular recognition of *M.tb.* However, the levels of genes related to cell growth, cell metabolism and hypoxia varied in the different subtypes. The varying immunological status of pulmonary macrophages suggests that they have either distinct origins or adapted to different microenvironment in TB patients [42]. Notably, caution should be taken to interpret the reports of novel cell subtypes, considering the possible false positive, such as doublets. The examples described above clearly demonstrate that scRNA-seq can greatly facilitate the identification of novel immune cell subtypes during infection.

#### 3.1.2 Profiling immune cell landscape during infection

The immune cells initiate host defenses against pathogenic infection. Profiling immune cell landscape characterize the overall immune cell compositions between physiological and infection conditions, which provides key information for understanding the pathogenic mechanism of infectious diseases. For instance, scRNA-seq analysis has divided the peripheral blood mononuclear cells (PBMCs) from HIV-1 envelope vaccinated neonatal and adult monkeys into 4 groups: B, T, NK, and monocyte [43]. It further demonstrated that each of the 4 cell clusters showed different patterns between neonatal and adult monkeys (Fig. 2B) [43]. A significant increase in the ratio of activated B cells was found in neonatal monkeys, indicating that the neonatal immune system produces a stronger protective response than that of adult monkeys during HIV infection [43]. This result also suggest that the expansion of activated B cells may play a vital role in controlling HIV infection [43]. Similarly, Park et al*.* reported that NK cells transformed into innate lymphoid cell 1 (ILC1)-like cells after *Toxoplasma gondii* infection via scRNA-seq analysis. The gene expression profile of ILC1-like cells differs from that of ILC1 cells. It maintains the ability of the ILC1 cells to produce IFN-γ but not TNF-α. Considering the fact that ILC1-like cells exist *in vivo* during and after infection, it is speculated that they play a protective role even after the parasite clearance [44]. Likewise, immune landscape profiling after mouse cytomegalovirus (MCMV) infection by scRNA-seq illustrated the differentiation trajectory of plasmacytoid dendritic cells (pDCs) during MCMV infection [45]. Zhang’s group developed the Single T-cell Analysis by RNA-seq and TCR Tracking (STARTRAC) method to reveal the dynamic population changes of 20 T-cell subtypes, each with different functions, in colorectal cancer [46]. scRNA-seq has also been applied to monitor the peripheral immune cell landscape changes in corona virus disease 2019 (COVID-19) patients [47], [48]. It was discovered that a series of changes occurred in peripheral immune cell landscape of COVID-19 patients, which included lymphopenia, T cell exhaustion and expanded myeloid compartment and plasmablasts [47]. Similarly, another study reported expanded cytotoxic effector T cell, including CD8^+^ effector-GNLY (granulysin), CD4 + effector-GNLY and NKT CD160, in convalescent patients [48]. Thus, scRNA-seq can identify changes in immune cell landscape during infection and reveal the unique role of these populations, shedding lights on the mechanisms of pathogenesis.

#### 3.1.3 Detecting changes of inflammatory responses

Inflammatory factors are mainly secreted by stimulated immune cells, and have various biological functions, including regulating innate and adaptive immune response, recruitment of immune cell, etc. Monitoring changes of inflammatory responses and identifying the key inflammatory factors following an infection are imperative for understanding the disease pathogenesis as well as developing novel treatment strategies. Using scRNA-seq analysis, Zhang et al*.* found varying production of various inflammatory factors in the lungs of mice infected with IAV (Fig. 2C). The first 3 days-post-infection (dpi), pro-inflammatory factors such as Tnf-α and Ccl3, were primarily produced by a special subset of *PD-L1 +* neutrophils. By 7 dpi, the virus is almost entirely cleared and chemokines including Ccl5 are primarily secreted by *Pf4 +* macrophages [49]. In COVID-19 assessed by scRNA-seq, SARS-CoV-2 infection induced IL-1β and TNF-α production may promote mucin secretion from club cells, likely contributing to acute respiratory distress syndrome (ARDS) [50]. These studies showed that application of scRNA-seq technology can facilitate the studying of changes in inflammatory factors secretions over the course of infection, revealing important cellular and molecular pathogenic mechanism.

#### 3.1.4 Identifying immune signaling pathways for differentially expressed genes during infection

scRNA-seq can not only identify the transcriptomic differences, but also map the cellular interacting networks. For instance, in one study aiming to understand B cell immunity against an influenza vaccine using scRNA-seq, it has been shown that a fraction of peripheral memory B cells were activated the vaccination, while other memory B cells remained inactive [51]. Expression differences in 172 genes have been found between activated and inactivate memory B cells, revealing the signaling pathways relevant to the host defense against influenza virus [51]. In another COVID-19 study, Wang’s group recently found the disease was associated with expression of multiple cytokines and the activation of inflammation-related signaling pathways in various activated cell subsets via scRNA-seq [52]. e.g., NK cells from healthy controls express genes related to antioxidant damage signaling pathways, while NK cells from COVID-19 patients show high expression of inflammation-related signaling pathways (Fig. 2) [52]. Similarly, using GO analysis of divergent genes, Zhang et al*.* showed that a subset of infected neutrophils was associated with pro-inflammatory response and neutrophil chemotaxis [49]. Thus, scRNA-seq enables the identification of immunological pathways that correspond to the differences in host gene expression throughout the course of an infection. This will deepen our understanding of the immunological processes occurring throughout the course of infection and assist the development of therapeutics for patients in different stages of infection.

### 3.2 Study the host-pathogen interaction

#### 3.2.1 Identifying susceptible cell types

**Fig. 3:**
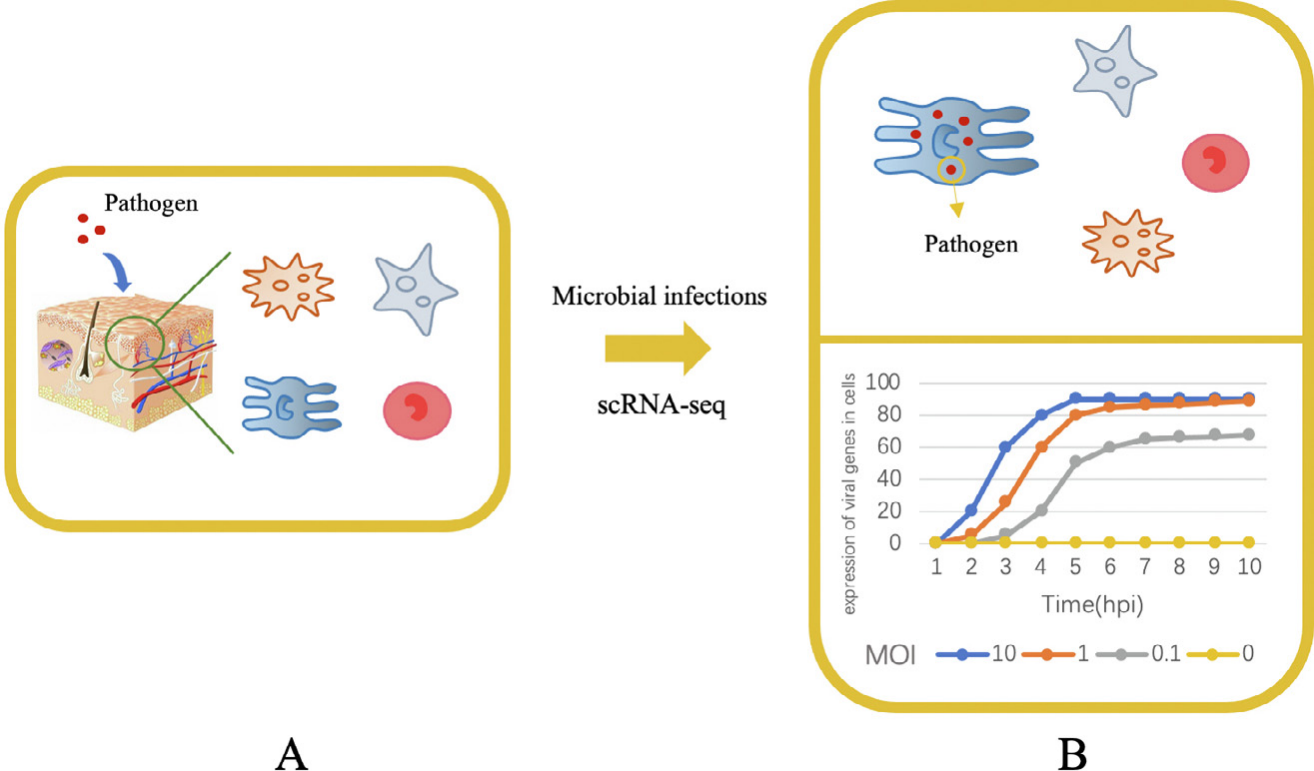
Study the host-pathogen interaction. (A) Identifying susceptible cell types; (B) Studying infection dynamics. Each curve represents the expression of viral genes contained in cells at varying levels of MOI.

For a specific pathogen, the identification of susceptible cells is crucial for understanding the pathogenic mechanism. For example, using scRNA-seq and cluster analysis, Steuerman et al*.* identified 5 types of immune cells and 4 types of non-immune cells in sorted CD45^+^ and CD45^-^ cells isolated from the lungs of both wild type and *Irf7* knockout mice infected with influenza virus. Further analysis revealed that the content of viral mRNA in epithelial cells was significantly higher than that of other cell types (Fig. 3A) [53]. The higher viral load presented a greater possibility of the virus colonizing and spreading between epithelial cells. Angiotensin-converting enzyme 2 (ACE2) has been demonstrated to be the crucial receptor for SARS-CoV-2, and cells expressing high levels of ACE2 are more susceptible for SARS-CoV-2 [54], [55]. By scRNA-seq, Zhang’s group recently reported that the SARS-CoV-2 receptor ACE-2 is primarily expressed in 8 cell types including lung alveolar type II cells, which has potential viral tropism [56]. Similarly, ACE2 mRNA has been found throughout the airway and shown to be highly expressed in proximal segments. In addition, *ACE2* mRNA expression is much higher in the constricted airways of smokers [57]. Except ACE2, TMPRSS2 is another factor for cell entry and spread of SARS-CoV-2 [58], [59]. The SARS-CoV-2 could not transmit vertically during pregnancy, likely due to negligible co-expression of ACE2 and TMPRSS2 in placenta cells [60]. These results, revealed by scRNA-seq, both clarified key factors for viral entry and identified susceptible cell types. In another example, Ben-Moshe et al*.* developed a new algorithm based on scRNA-seq to find the specific cell types associated with different microbial infections, demonstrating the capability to predict infectious disease risk and consequences [61]. *Salmonella* infected patients have fewer NKT cells than healthy controls, indicating that NKT cells have a protective effect [61]. In addition, Bost et al*.* developed a new tool called Viral-Track, which aims to map viral RNA directly from scRNA-seq data. Viral-Track maps scRNA-seq data to a large database of known viral genomes, providing precise annotations of cell types associated with viral infection [62]. They report that SARS-CoV-2 infection has a greater impact on the immune system of severely infected patients, inducing an altered cytotoxic response in CD8^+^ T cells. They also show that the virus primarily infects epithelial and macrophage subsets [62]. scRNA-seq can provide crucial information about cell types susceptible to infection which will help the development of strategies for intervention.

#### 3.2.2 Studying infection dynamics

It is important to understand infection dynamics, which focus on how pathogen proliferate and disseminate *in vivo* and how these link to pathogenesis. These studies may facilitate the development of personalized therapies for patients at different courses of infection, which will enable hierarchical diagnosis/treatment and shortening treatment time. However, some of the obstacles in these studies are the heterogeneity issues, manifested in both host cells and microbial populations, which determines how the host and pathogen interact [63]. The dissemination of bacteria between different immune cells, pathogen recognition by those immune cells, and pathogen mediated immune cell death could be clarified using scRNA-seq. For example, Romas et al*.* analyzed the dynamics of the interaction between IAV and respiratory epithelial cells at the single cell resolution in the early stage of infection and found that the multiplicity of infection (MOI) of viruses infecting host cells had a great impact on host innate immunity [64]. High MOI leads to increase intracellular viral mRNA (Fig. 3B) and stronger antagonistic effects on innate immune responses, such as the suppression of IFN production [64]. It was also found that the early innate immune response caused by low dose virus infection had a protective effect on bystander cells, which could block the further spreading of the virus [64]. Similarly, Zanini et al*.* studied the dynamics of the interaction between flavivirus and host cells by virus-inclusive single cell RNA-seq, and identified several host factors specifically related to flavivirus infection, including proteins involved in endoplasmic tranlocon, signal peptide processing and membrane trafficking [65]. The dynamics of SARS-CoV-2 infection have also been addressed by scRNA-seq. Compared with patients with moderate and severe diseases, studies reveals great differences in the immune cell composition at early and late convalescent stages [52]. Therefore, scRNA-seq can discern the systematic effects of the interaction between pathogens and host cells, revealing varying effects caused by varying viral loads and stages of the host’s immune response.

### 3.3 Studying immune repertoire

**Fig. 4:**
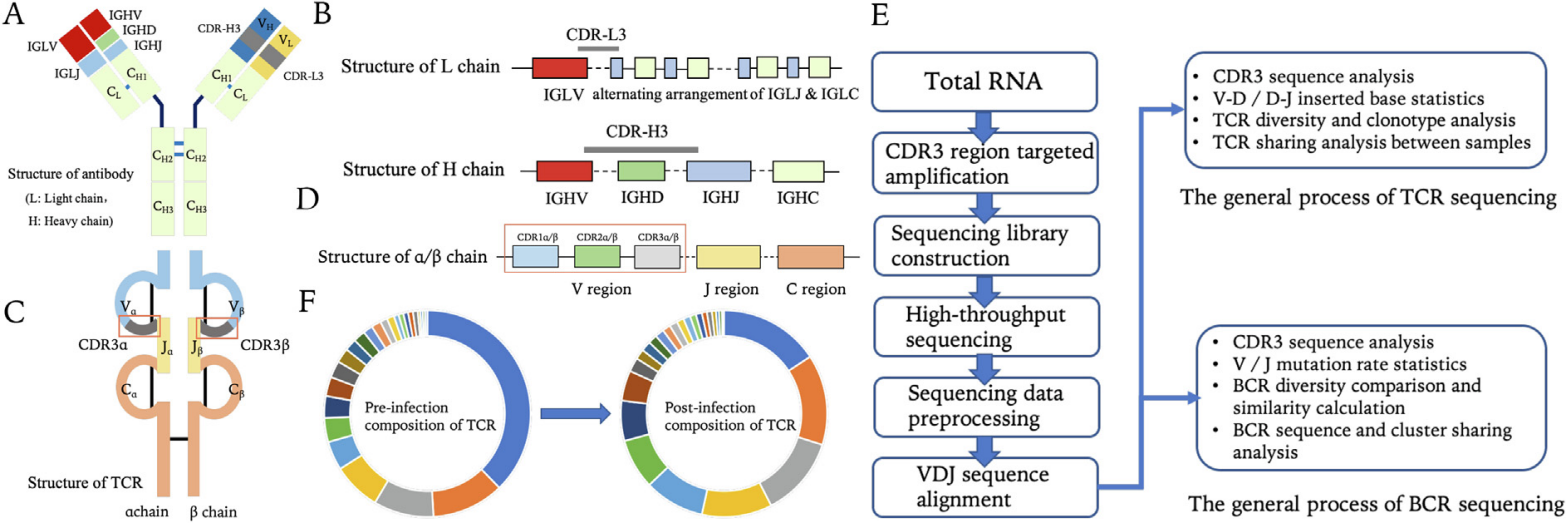
TCR and BCR sequencing. (A) Antibody Structure; (B) BCR gene structure, including IGLV, IGLJ, IGLC, IGHV, IGHD, IGHJ, IGHC (IG-, Immune Globulin-, immunoglobulin, LV / LJ / LC / HV / HD / HJ / HC indicates the light chain variable region / light chain binding region / light chain constant region / heavy chain variable region / heavy chain multivariable region / heavy chain binding region / heavy chain constant region respectively). Colors reflect structural regions shown in A and B; (C) Structure of the TCR; (D) TCR gene structure. Colors reflect structural regions shown in A and B; (E) Standard procedure for BCR / TCR sequencing; (F) Changes in TCR composition before and after infection.

Immune repertoire refers to all B cells and T cells with distinct antigen specificity at any given time. It has important applications in identifying potent neutralizing antibodies and testing the efficacy of vaccinations. B cell receptors (BCR) on the surface of B cells recognize and bind antigen. The BCR consists of two heavy chains (H chain) and two light chains (L chain). The H chain is composed of four parts: the variable region (V region), the diversity region (D region), the joining region (J region) and the constant region (C region). The L chain is composed of three parts: V, J and C region. Similarly, TCR on the surface of T cells is composed of TCR α and β chains with V, J and C region as well [66], [67], [68]. The diversity of the V region, along with different VDJ rearrangement, and the deletion or insertion of nucleotides at the junctions of different gene fragments create BCR and TCR diversity. The structure of the corresponding BCR gene, TCR, and coding gene are shown in Fig. 4. The immune repertoire analysis includes CDR3 sequence analysis, single cell VDJ statistics, TCR or BCR diversity analysis, and TCR or BCR sharing analysis between samples (Fig. 4E). scRNA-seq can accelerate the identification of neutralizing antibodies with potent therapeutic and prophylactic effects, which are vital for the prevention of the emerging or pandemic infectious diseases. For example, Xie’s group sequenced the antigen-enriched B cells from the plasma of convalescent patients with COVID-19 using scRNA-seq and BCR sequencing, and recovered 14 highly active virus neutralizing antibodies [69]. *In vivo* studies confirmed that one neutralizing antibodies, BD368-2, showed strong potential as a therapeutic [69]. scRNA-seq can also be applied to monitor the T cell responses after infection or test the efficacy of vaccination. Liao et al*.* recently performed the scRNA-seq and single cell TCR-seq on the bronchoalveolar lavage fluid from COVID-19 patients. They reported a greater increase of clonally expanded CD8^+^ T cells in mildly infected patients than that of severely infected patients [70]. In another study, TAK-003 (a potential recombination tetravalent DENV vaccine) was found by scRNA-seq analysis of PBMC from patients post immunization and proven to be an excellent candidate eliciting the potent and durable T cell response [40]. Another group used single-cell BCR & TCR sequencing, revealing different types of T cell clonal expansion (Fig. 4F) and a unique composition of BCR immunoglobulin in convalescent COVID-19 patients [52]. Moreover, unique combinations of BCR genes is associated with specific pathogen infection. For instance, VDJ in severe COVID-19 patients had a different pattern when compared with health controls, indicating specific BCR-VDJ rearrangement in severe COVID-19 [48]. Thus, scRNA-seq, combined with BCR /TCR sequencing, could provide important insights into the adaptive immune responses against infection, and accelerate the development of neutralizing antibodies and specific vaccines. This is vital for the prevention of emerging or pandemic infectious diseases.

### 3.4 Biomarker discovery for infectious diseases

A biomarker refers to robust biochemical or cellular changes during certain biological process and has been widely applied in diseases diagnose and biomedical research [71]. Currently, concrete biomarkers for infectious diseases are limited, and further exploration is still required. scRNA-seq greatly facilitates the identification disease-related biomarkers. For example, Zanini et al. performed scRNA-seq on PBMCs from patients with dengue disease and found that the expression of *MX2* in naive B cells and the expression of *CD163* and *IFIT1* in CD14^+^CD16^+^ monocytes were significantly up-regulated before severe dengue disease developed [72]. These upregulated genes may be used as biomarkers to predict the progress of dengue disease in the future [72]. Similarly, by comparing single cell transcriptomes of PBMCs between sepsis patients and healthy controls, Reyes et al*.* found that placenta-associated 8 (*PLAC8*) and clusterin (*CLU*) mRNA expression level in a monocyte subgroup called MS1 (monocyte states 1) were significantly higher in sepsis patients [73]. Another study refers to the discovery of biomarker for active TB. Cai et al. collected the PBMCs from healthy people, latent TB patients and active TB patients for scRNA-seq. They found that NK cell (CD3^-^CD7^+^ GZMB^+^) subsets were depleted in active TB patients, and this cell population recovered in cured TB patients, suggesting that this NK subset could be a marker to identify active TB patients and monitor treatment response [74]. These results were consistent with a multi-cohort research performed during tuberculosis infection [75], which reported that NK cell levels decreased in progression from latent infection to active TB, while NK cell number significantly increased at the end of treatment [75]. Hence, NK cell could be a novel biomarker for active TB. These studies indicate scRNA-seq can be applied to monitor gene expression patterns related to specific infections, which can reveal candidate biomarkers for disease diagnosis and prognosis.

## 4 Perspective

### 4.1 The developmental trend of scRNA-seq

Since most of the information acquired by bulk RNA-seq can also be acquired by scRNA-seq, scRNA-seq shows great advantage in solving complex biological and clinical problems from a systematic approach. With the emergence of several commercial platforms, scRNA-seq is being increasingly utilized. The average cost of scRNA-seq has fallen to below $1 per cell [11], [23], including the cost for library construction and sequencing, continuous improvement in cost-efficiency will encourage more application of scRNA-seq in developmental biology [76], [77], oncology [78], [79], neuroscience [31], etc. The combination of live cell sorting with scRNA-seq greatly improves sample viability, eliminating concern for most clinical tissue samples. Technical requirements for scRNA-seq data analysis are also decreasing, as the number of R language packages for scRNA-seq, such as Seurat and Monocle, and commercial data analysis platforms are growing rapidly. Researchers from Theis lab have summarized current mature practices for each step in scRNA-seq analysis [80], including linked inference of genomics experimental relationships (LIGER) (a tool to approximate the original data and to share factors across various datasets) [81] and Multi-omics factor analysis (MOFA) (a tool for analyzing matched data) [82]. Online servers could make the analysis of scRNA-seq data simpler and more comprehensive, such as alona and scQuery [83], [84]. All these developments may gradually push the replacement of traditional bulk RNA-seq by scRNA-seq. However, in some special cases, bulk RNA-seq still has irreplaceable advantages. For instance, certain types of cells are fragile during sample preparation, preventing them from being sequenced, whereas the bulk RNA-seq could handle it accurately. In addition, requirement of single-cell suspension plus high cell viability also prevent the application of scRNA-seq for most plant or special animal tissue samples. With recent development, bulk RNA-seq data can now be utilized to infer scRNA-seq data, such as SCRABBLE algorithm [61], [85], [86], [87]. Similar to bulk RNA-seq, microarray can detect the differential expression of RNA, but it depends on a hybridization generated signal. Although the sensitivity of gene expression score detected by microarray is reduced compared with bulk RNA-seq and scRNA-seq, microarray are still very popular in clinical research because of its simple use and fast results [88].

At present, mainstream commercial platforms for scRNA-seq include *10X Genomics* and *BD Rhapsody*. The sample processing time of both platforms is within 30 min; and the entire process is integrated, including library construction, sequencing and data analysis (optional). In addition, the ratio of captured doublets by these two platforms is relatively low. While both are widely used in research fields such as immunology, oncology and neurology, there are still some minor differences between these two platforms. The sequencing throughput of *10X Genomics* is higher than that of *BD Rhapsody*, and *10X Genomics* has a unique technology that can complete spatial transcriptome sequencing [8], [89]. More importantly, it’s easier to operate than the *BD Rhapsody*. On the other hand, the plate-based *BD rhapsody* requires fairly low concentrations of cell suspensions, while a higher concentration is necessary for microdroplet-based systems, like the 10X Genomics [10]. In addition, *BD Rhapsody* provides quality control of the samples via direct visual inspection of cartridge contents and each microwell [10], [90]. Regarding cost efficiency, *BD Rhapsody* beads can be retained for a longer period, which enables the construction of multiple sequencing libraries from subsample beads. For now this cannot be achieved by *10X Genomics* [10]. Researchers should consider the various scope of applications, cost efficiency and complexity of work flow between these two platforms, when determining which to be applied according to specific purposes.

### 4.2 Emerging methods based on scRNA-seq

Based on scRNA-seq, several novel single-cell multi-omics technologies have emerged. For example, performing scRNA-seq with oligo-nucleotides labeled antigens or antibodies, have allowed simultaneous detection of gene expression, VDJ, antigen specificity, and cell surface markers [91], [92]. These novel immune profiling techniques have been combined to characterize the gene expression and TCR repertoire in breast cancer and paracancerous tissues, blood and lymph nodes. Detailed immune cell maps of multiple immunophenotypes in tumor microenvironment have been drawn via this joint sequencing method, which has deepened our understanding of tumor cell heterogeneity [93].

**Fig. 5:**
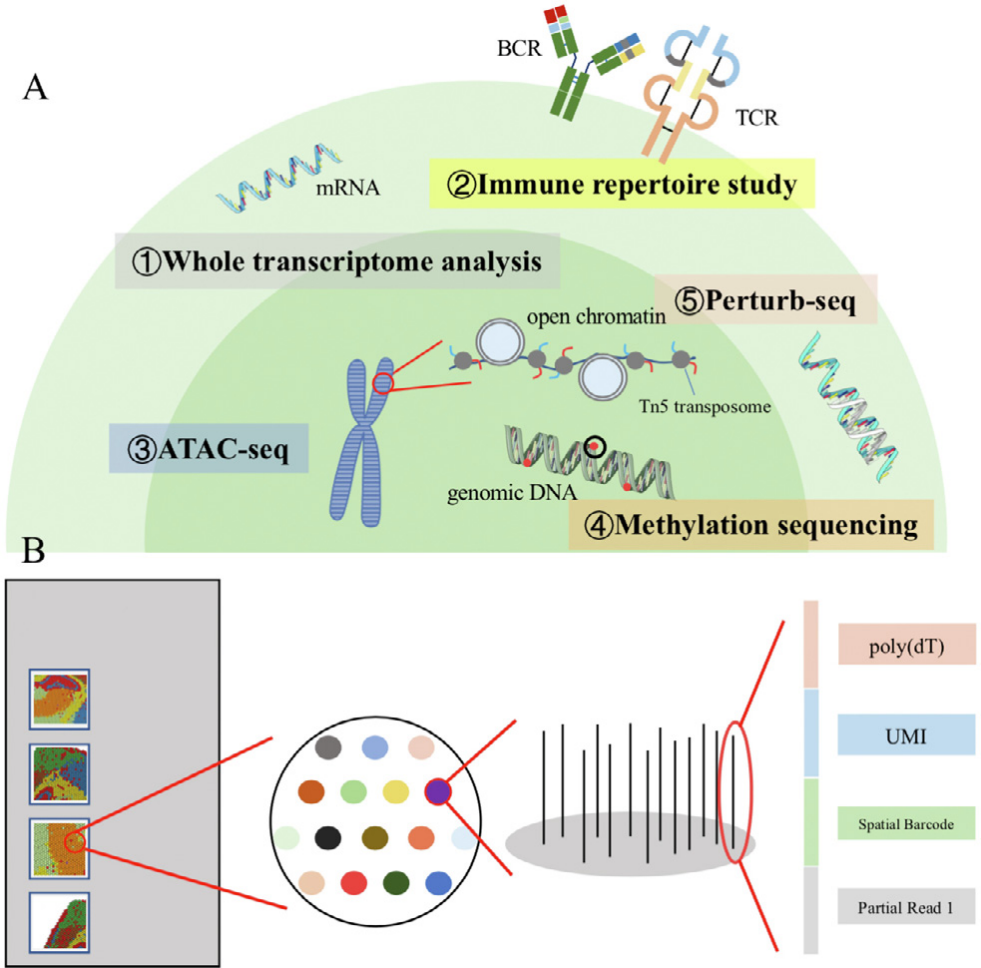
Several emerging single-cell sequencing methods. (A) Including ATAC-seq, whole transcriptome analysis, immune repertoire study, CRISPR-I and methylation sequencing; (B) Schematic diagram of a spatial transcriptome sequencing chips. There are four chips for capturing single-cell mRNA on a slice. Each spot in the chip contains an oligonucleotide sequence, which includes poly (dT), UMI, spatial barcode and partial read 1. Partial read 1 includes 22nt for Illumina sequencing, spatial barcode includes 16nt 10X barcode, and UMI includes 12nt unique molecular identifier.

The newest spatial transcriptomic technology can trace the gene expression information of cells to the original spatial location within the tissue (Fig. 5A). Spatial transcriptome techniques have already been used in cancer related studies. Moncada et al*.* utilized spatial transcriptome and scRNA-seq to analyze the spatial distribution of immune and non-immune cells in sections of pancreatic cancer, the result of which indicated inflammatory fibroblasts were enriched near tumor cells with high expression of stress response genes [94]. Recently, online analysis platforms for spatial transcription data have been established [95]. With those emerging tools, the visual interpretation of spatial data and annotations for spatially differentially expressed genes and enrichment analysis can be achieved [95]. Besides these advantages, some limitations still need to be addressed. Since spatial transcriptomic technology is built on the chip, the size of measured tissue section is determined by the chip size, and the transcripts diffusion on the chip is inevitable [94]. Additionally, each single measuring spot on the chip actually captures more than a single cell, which will affect the subsequent clustering analysis [7]. Currently, several spatial transcriptomic techniques are developed, including Slide-seq [96], MERFISH (multiplexed, error robust fluorescent in situ hybridization) [97] and seqFISH (sequential fluorescent in situ hybridization) [98]. Those emerging technologies will promote the discovery of medical biology to a new level.

In eukaryotes, DNA is combined with histones to form nucleosomes, which are further folded and compressed to form chromatin. Both DNA replication and transcription require opening the tight chromatin structure to allow regulatory factors to bind to the DNA. This property is called chromatin accessibility. Assay for Transposase-Accessible Chromatin using sequencing (ATAC-seq) is a integrative epigenomic analysis method that based on in vitro relocation of sequencing adaptors into native chromatin (Fig. 5A) [99]. ATAC-seq could find target genes regulated by transcription factors, or identify regulatory elements required during gene expression. In addition, Chromosome Conformation Capture (3C) technology and its derivatives, such as Hi-C, ChIA-PET, enables high-throughput quantitative sequencing to analyze the three-dimensional structure of the nucleus with superior resolution [100], [101], [102].

DNA methylation is an important epigenetic marker (Fig. 5A). It is closely related to gene imprinting, stem cell differentiation, and tumor occurrence / development. Obtaining the single-cell methylome is highly significant in the study of epigenetic spatio-temporal specificity. Currently, detection of DNA methylation at single-cell resolution is possible, although it cannot completely cover all CpG positions [103], [104]. Angermueller et al*.* recently developed a calculation method DeepCpG, which is based on a deep neural network strategy and can fill gaps left by previous methods [105].

Recently, another interesting development called “direct-capture perturb-seq” was reported. This combines clustered regularly interspaced short palindromic repeats (CRISPR) screening with scRNA-seq (Fig. 5A). By using this technique, gene expression changes caused by individual sgRNA and the sgRNA itself can be measured with single cell precision. This study revealed epistatic interactions between cholesterol biogenesis and DNA repair were revealed [106]. This method combines two important technologies and enables alternative ways to probe the relationship between genes and signal pathways. Therefore, multi-omics analysis methods examine individual cell by various dimensions, and enable comprehensive exploration of cellular characteristics and functions. [107], [108].

### 4.3 Challenges in scRNA-seq

Some challenges still exist for scRNA-seq. Since bacterial mRNA and some viral mRNA do not have a 3′ poly-A tail, their mRNA cannot be studied using current scRNA-seq techniques. In addition, doublets identification remains problematic during data analysis [109]. Since doublets have the mixed transcriptome from two cells, it may cause false positive discovery of novel cell subtypes. However, analyzing the doublets may also reveal the authentic cell-to-cell interaction information. Indeed, there is higher percentage of doublets in the blood of patients with tuberculosis and dengue disease [110]. These doublets often consist of T cells and monocytes adhering to each other [110]. Some bioinformatic tools have been created, such as SOLO (a semi-supervised deep neural network model to identify doublets) [111], Scrublet [112] and DoublettFinder [113]. To avoid losing crucial information relevant to the disease, careful consideration should be taken when deciding whether doublets should be excluded from the sequencing analysis.

The results of multi-omics sequencing involve the integration of heterogeneous data from multiple source. For example, when different data from scRNA-seq and ATAC-seq are integrated for analysis, the inconsistency of data characteristics cannot be dealt properly. It is necessary to use algorithms to integrate data from various data source, such as Specific Representation Learning (an analysis strategy that integrates scRNA-seq data sets based on common mutation sources to identify shared populations across data sets and perform downstream comparative analysis) [114], [115], MOFA (a computational method that discovers the cooperation between methylation sites and transcriptome) [82], and Manifold Alignment to CHaracterize Experimental Relationships (MATCHER) (a method that utilizes multiple alignments to infer single-cell multi-omics profiles between transcriptome and epigenetic data achieved on various cells of the same type) [107], [116], [117]. Among these methods, machine learning is the leading choice with the help of computer sciences [107]. Researchers could use computer programming methods to simplify the complexity between different data sources. By using these programs, these data can be integrated according to the key characteristics. At the same time, the high-speed computing capabilities could capture and predict data that cannot be manually identified. In short, integration of multi-omics could allow researchers to understand how several realms interact. Through high-throughput sequencing of various omics and data integration research, it is possible to comprehensively and systematically understand the interrelationship of multiple substances in the fields of basic research, clinical diagnosis, and drug development. The discovery of valuable immunological information from scRNA-seq results need the collaborative effort of not only comprehensive immunological knowledge, but also intensive data analysis abilities.

Missing values caused by the failure of RNA amplification in cells is a significant problem that exists in scRNA-seq, making it technically impossible to judge whether the gene was missing or not expressed. Imputation is an appropriate method to address the problem [118], [119]. For instance, by taking advantage of scRNA-seq, Ben-Moshe et al*.* exploited a deconvolution algorithm to infer cell-type specific infection responses from bulk RNA-seq data, and this algorithm could provide a predictive power for TB progression [61]. Currently, some tools have been developed to accurately impute dropouts in scRNA-seq data, such as single-cell analysis via expression recovery (SAVER) [118], scImpute [87] and Markov affinity-based graph imputation of cells (MAGIC) [120].

Besides Smart-seq, the sequencing depth of most scRNA-seq technologies is about 1 million reads, this leaves much room for improvement in future [11]. scRNA-seq for lncRNA is currently possible, and sequencing methods for other non-coding RNA are under development [121].

scRNA-seq has greatly improved our understanding about the heterogeneity in various biological process, and has been involved in many breakthroughs throughout immunology, oncology, and developmental biology. With the combination of scRNA-seq and multi-omics analysis, the pathogenic mechanism of various infectious diseases could be quickly and systematically elucidated, significantly propelling vaccine development in the near future.

## Declaration of Competing Interest

The authors declare that they have no known competing financial interests or personal relationships that could have appeared to influence the work reported in this paper.
